# Case report: MELAS and concomitant presumed antiphospholipid antibody syndrome in an adult woman

**DOI:** 10.3389/fneur.2022.1043695

**Published:** 2022-12-14

**Authors:** Sirisha Nouduri, Rajiv Padmanabhan, Richard Hicks, Mary-Alice Abbott, Dennis O'Brien, Gottfried Schlaug

**Affiliations:** ^1^Department of Neurology, Baystate Medical Center, University of Massachusetts Chan Medical School—Baystate, Springfield, MA, United States; ^2^Department of Radiology, Baystate Medical Center, University of Massachusetts Chan Medical School—Baystate, Springfield, MA, United States; ^3^Department of Pediatrics, Baystate Medical Center, University of Massachusetts Chan Medical School—Baystate, Springfield, MA, United States

**Keywords:** MELAS, antiphospholipid antibodies, anticardiolipin antibodies, heteroplasmy, MRI, MRS

## Abstract

Mitochondrial encephalomyopathy, lactic acidosis, stroke-like episodes, and other features (short stature, headaches, seizures, and sensorineural hearing loss) constitute characteristics of MELAS syndrome. MELAS is a rare condition due to mutations in maternally inherited mitochondrial DNA with levels of heteroplasmy possibly related to late adulthood presentation. A previously reported MELAS case coexisted with presumed Antiphospholipid Antibody Syndrome (APLAS), but the connection between MELAS and a potential APLAS is unclear. A 29-year-old woman presented with mild right-sided sensorimotor symptoms and mixed aphasia in November 2021. She presented again in May 2022 for unrelenting headaches and was found to have a new right hemisphere syndrome with mild left-sided sensorimotor symptoms, hemineglect, and anosognosia. Characteristic lab and imaging studies were obtained. During the first presentation (October 2021), the discovery of anticardiolipin IgM antibodies (aCL) (and their replication 3 months later) led to a diagnosis of APLAS, and Warfarin was initiated. During the second admission (May 2022), a new stroke-like lesion on the right hemisphere with characteristic features not suggestive of ischemia was detected, which led to a diagnosis of MELAS (m3243A > G mutation). Although MELAS and APLAS could co-exist, alternatively, it is possible that antiphospholipid antibodies might be generated when the strongly anionic Cardiolipin-Hydroperoxide from the inner mitochondrial membrane is exposed to immune component cells upon cell lysis. Thus, the presence of aCL in patients with stroke-like lesions might masquerade as an APLAS, but should probably be questioned if only aCL are repeatedly found and imaging findings are not characteristic for ischemic lesions.

## Case description

**Figure 1:**
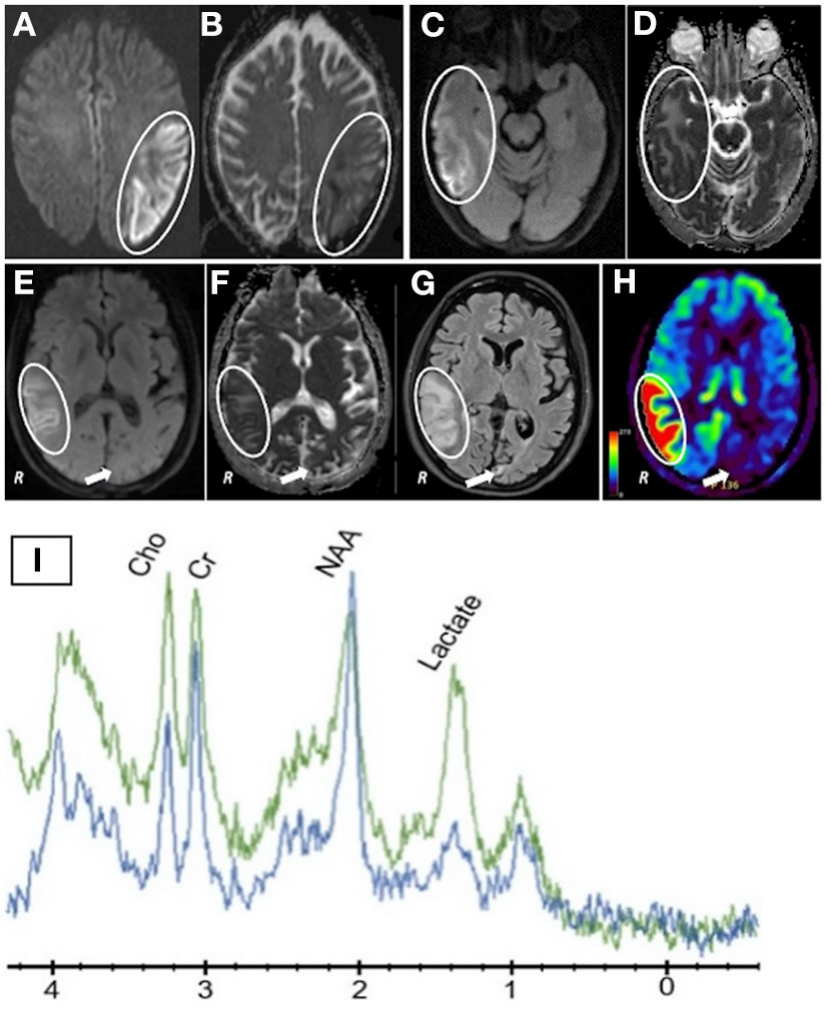
**(A)** DWI scan after first presentation (November 2021) showing a bright signal mainly in cortical regions; **(B)** Calculated ADC map of the first DWI scan shows minor alterations with reductions (cortex) and slight increases in the subcortical white matter; **(C,E)** DWI scan of new temporo-parietal lesion (second MRI scan); **(D,F)** Calculated ADC map of the second DWI scan (May 2022) revealing a similar pattern as the first MRI scan; **(G)** FLAIR scan (May 2022) of new temporo-parietal lesion showing prominent and extensive signal abnormalities, but only speckled lesions (arrow) in the previous acute left hemisphere lesion seen in November 2021; **(H)** Arterial Spin Labeling (ASL) blood flow study displaying hyperperfusion in the new right temporo-parietal lesion and hypoperfusion in the residual chronic lesion on the left hemisphere (arrow); **(I)** MR Spectroscopy obtained during the second presentation (May 2022). The green graph represents an ROI within the new right temporo-parietal region and the blue graph representing an ROI in a control, unaffected region of the brain. A large difference in the Lactate peak between affected and unaffected brain region can be seen.

A 29-year-old woman, not on any cardiovascular or antipsychotic medications, presented in November 2021 with what appeared to be her first left temporo-parietal stroke. Initially, it was thought that the lesion corresponded to the inferior division of the middle cerebral artery (MCA) (Figure 1A), but this was later revised and thought to not confirm to a particular vascular territory. There was no occlusion of extra- or intracranial vessels on CT angiography (CTA). Retrospective analysis of the Apparent Diffusion Coefficient (ADC) map showed only a mild reduction in the cortex by about 10% of normal (Figure 1B) with a possible small increase in diffusivity in the subcortical white matter region. These alterations in the diffusivity of water protons are not consistent with what is typically seen in true ischemic tissue where reductions of about 30–50%, compared to normal, have been reported in the acute stroke phase (1, 2). Further work-up revealed an abnormal level of anticardiolipin IgM antibodies (aCL IgM) with 29.4 MPL (normal <12.5 MPL), no antibodies against Beta2-Glycoprotein1 (B2GP1), and negative Lupus Anticoagulant. A CTA of her chest and a repeat transesophageal echo (TEE) ruled out a Patent Foramen Ovale (PFO) or a pulmonary arterio-venous fistula. Her neurological examination was characterized by a mild right sensorimotor impairment, mixed aphasia, and some right visual hemifield impairment. Two days after onset of her initial stroke symptoms, her blood Lactic Acid (LA) level was found to be elevated at 2.4 mmol/L, but then normalized to 1.9 mmol/L one day later (normal 0.5–2.2) after intravenous fluids were administered. Five days after symptom onset, she developed right upper extremity rhythmic clonic activities (presumably an Epilepsia Partialis Continua) and was started on Levetiracetam. This medication was tolerated well by the patient and controlled the rhythmic clonic activity. There was no personal or familial history of seizures. Repeated APLAS antibody testing (3 months later) revealed an increasing titer of aCL IgM, now at 45 MPL (Normal <9 in our lab), but again no aCL IgG antibodies, B2GP1 IgG/IgM antibodies, or Lupus Anticoagulant. Warfarin was started as recommended by hematology for the presumptive diagnosis of APLAS. Since the patient had been complaining of decreased hearing for a while, a hearing test in March 2022 revealed sensorineural hearing loss which was not communicated to her Neurologists until the second admission in May 2022.

About 7 months after the initial presentation, she presented again with unrelenting holocranial headaches. Head CT and MRI confirmed a new right temporo-parietal lesion extending beyond one single vascular territory (Figure 1C). On neurological exam, she had a right hemisphere syndrome with a mild sensorimotor dysfunction of the left upper and lower extremities, inattention to the left and anosognosia. Again, detailed MR imaging and analysis were not typical for ischemia. Although the DWI was bright, only minor alterations in either direction in the calculated ADC values were seen compared to normal brain tissue (Figure 1D). Of note, only small speckled residual T2 lesions were seen in the previous large left hemisphere lesion from November 2021 (Figure 1G, arrow). Surprisingly, new Blood Flow MR images revealed hyperperfusion (Figure 1H, circle) in the area of the new lesion on the right (Figures 1E,F). MR spectroscopy within the right hemispheric lesion showed a high tissue lactate level with slightly reduced NAA peaks compared to an unaffected region in the right frontal lobe (Figure 1I). These imaging findings are similar to reports of MELAS patients (3–5). The presence of high lactic acid levels could have triggered the regional hyperperfusion. The MR blood flow image showed also some heterogeneity reflecting old (Figure 1H, arrow) and new lesions (Figure 1H, circle). A CTA again showed open vessels. Her serum Lactic Acid levels were elevated at 3.6 mmol/L (upper normal is 2.2).

Given two episodes of stroke-like events, lactic acidosis (without any evidence for infection or sepsis), short stature (5 feet), sensorineural hearing loss, headaches, and focal seizures, she was presumed to have MELAS (6). Genetic testing confirmed the m3243A > G point mutation with a heteroplasmy of 24.8% measured in blood cells (7). She was treated with L-Arginine infusions (0.5 g per kg bodyweight) daily for 5 days, started on Co-Enzyme Q10 (200 mg per day, 2 oral capsules), her Levetiracetam from the first admission was continued, and Warfarin was discontinued. Her headaches resolved and her neurological dysfunction slowly improved over several months. Medications were well-tolerated, but compliance was suboptimal at times given the patient's reluctance to accept a diagnosis of MELAS.

A few missed clues delaying the diagnosis of MELAS after the first presentation are worth discussing. The transient slight increase in lactic acid after the first stroke-like episode was misclassified as dehydration secondary to its rapid normalization after rehydration. Since the aCL IgM was the only antiphospholipid antibody to be positive on repeated testing, a presumptive diagnosis of APLAS was made, but as we discuss below, it should have been questioned. aCL IgM remains positive at her 11-month evaluation after the index event. Cardiolipin is known to reside in the inner mitochondrial membrane. If the mitochondrial membrane degrades as a result of oxidative stress and other factors, Cardiolipin hydroperoxide can become extracellular and promotes a strong immune responses (8). Cardiolipin hydroperoxide may not bind to β2GPI, as Cardiolipin conventionally does (9). It is an anionic and highly immunogenic phospholipid which has not been previously seen by immune competent cells which will launch a strong immune response. A persistent immune response due to the continuous release of anionic Cardiolipin might explain the long-lasting aCL IgM response in our patient as well as potentially others.

Therefore, we suggest that in younger adult patients presenting with strokes or stroke-like episodes with isolated aCL IgM, the possibility of late-onset MELAS should be considered. An APLAS diagnosis should be questioned until MELAS is excluded, especially given the therapeutic implications of life-long antithrombotics in young patients.

## Data availability statement

The datasets presented in this article are not readily available because of ethical and privacy restrictions. Requests to access the datasets should be directed to GS, gschlaug@gmail.com.

## Ethics statement

Ethical review and approval was not required for the study on human participants in accordance with the local legislation and institutional requirements. The patients/participants provided their written informed consent to participate in this study.

## Author contributions

SN, RP, RH, M-AA, DO'B, and GS: conception and design, acquisition of data, analysis and interpretation of data, drafting, and revising manuscript. All authors accept responsibility for the integrity of the data analyzed, contributed to the article, and approved the submitted version.
